# Revisiting the Carbon Footprint of Single‐Use Technologies in Biomanufacturing: A Bottom‐Up Analysis Reveals a Paradigm Shift

**DOI:** 10.1002/bit.70297

**Published:** 2026-07-05

**Authors:** Jan Reiners, Katharina Bruno‐Thakur, Daina Romeo, Weronika Kuśmierczyk, Ferdinand Stückler

**Affiliations:** ^1^ Roche Diagnostics GmbH Penzberg Germany; ^2^ F. Hoffmann‐La Roche Ltd. Basel Switzerland; ^3^ Lonza Basel Switzerland

**Keywords:** biotechnology, mathematical model, processing, single‐use technology, sustainability, unit operations

## Abstract

The biopharmaceutical industry increasingly relies on single‐use technologies (SUT) for operational flexibility. For a long time, SUT was considered more sustainable than stainless‐steel (SST) systems due to water and energy savings. This study re‐evaluates this paradigm via a detailed bottom‐up analysis of the SUT carbon footprint at the 2000 L scale. Our analysis, based on two real‐world facility case studies (a full‐SUT and a hybrid‐SUT model), shows that the CO2 footprint of SUT is significantly higher than previously assumed. This granular analysis, based on physical disassembly and updated “cradle‐to‐gate” factors, identifies filters and bags as key emission hotspots. A separate comparative analysis also shows that key SST process steps—favored by the progressive decarbonization of electricity grids—can now have a lower carbon footprint than their SUT counterparts. Our data demonstrates that an operationally optimized hybrid facility design, which combines SUT with SST, can significantly reduce plastic waste and associated emissions. These findings compel a reassessment of sustainability strategies in biopharmaceutical manufacturing and highlight the potential of fit‐for‐purpose hybrid models as an effective lever for reducing the ecological footprint.

**Abbreviations**
BOM: bill of materials
CIP: cleaning‐in‐place
CtG: cradle‐to‐gate
EoL: end‐of‐life
GHG: greenhouse gas
GMP: good manufacturing practice.
GWP: global warming potential
HVAC: heating, ventilation, and air conditioning
IEX: ion exchange chromatography
LCA: life cycle assessment
MSAT: manufacturing science and technology
NIIMBL: National Institute for Innovation in Manufacturing Biopharmaceuticals
PC: polycarbonate
PE: polyethylene
PP: polypropylene
PSU: polysulfone
PVC: polyvinyl chloride
SBTi: Scientific Based Target initiative
SIP: sterilizing‐in‐place
SST: stainless‐steel technology
SUT: single‐use technology
TPE: thermoplastic elastomer
UF/DF: ultrafiltration/diafiltration

## 1 Introduction

The biopharmaceutical industry's increasing adoption of single‐use technologies (SUT) offers significant operational advantages, such as faster change‐over, operational flexibility, and reduced cross‐contamination risk. However, this operational advantage comes with a significant ecological trade‐off: the generation of substantial amounts of plastic waste. Estimates suggest that large quantities of plastic waste are generated annually in the healthcare sector, a figure that continues to grow significantly (Li et al. 2025).

For a long time, the established paradigm held that producing biopharmaceutical drug substances in SUT, despite the waste issue, represented a more environmentally friendly alternative to reusable stainless‐steel systems (SST) (Ottinger et al. 2022). Seminal studies, such as that by Pietrzykowski et al. (2011), demonstrated that SUT drastically reduces the on‐site consumption of water and energy (typical Scope 1 and 2 CO2eq emissions according to the GCG protocol (Ranganathan et al. 2004) by eliminating the need for resource‐intensive cleaning‐in‐place (CIP)/sterilizing‐in‐place (SIP) operations, as well as less heating, ventilation, and air conditioning (HVAC) due to a smaller floor footprint. However, this advantage shifted the environmental burden from the manufacturing phase to other parts of the life cycle, particularly to Scope 3 emissions (emissions due to supply chain) arising from the continuous consumption of plastic materials and their disposal. For the pharmaceutical industry, Scope 3 emissions are dominant, often accounting for over 90% of the total carbon footprint and currently the main sustainability challenge (Booth et al. 2023).

Two crucial developments now fundamentally challenge this established paradigm. First, the progressive decarbonization of electricity grids has significantly mitigated the CO2 disadvantage of energy‐intensive processes in SST. While this physical grid transition reflects an irreversible global trend—with the International Energy Agency projecting that non‐fossil sources will supply nearly 60% of global electricity by 2035, even with increasing demand (IEA 2025), prominent regional examples already demonstrate the rapid speed of this shift today. For example, since 2011, the share of electricity generated by low‐carbon sources has risen from 51% to 71% in 2024 in the EU (Ember et al. 2025). Similarly, in California, where many biotechnology facilities operate, this share exceeded 100% of demand for a record 98 of 116 days from late winter to early summer, 2024 (Jacobson et al. 2025).

Furthermore, for manufacturing hubs where local physical grids are still transitioning, the biopharmaceutical industry increasingly drives immediate decarbonization via market‐based accounting. By leveraging Power Purchase Agreements (PPAs), green tariffs, and on‐site photovoltaics, global production networks—with leading multinational organizations already sourcing 100% sustainable electricity across all worldwide operations—effectively decouple their Scope 2 emissions from local grid carbon intensities. Consequently, low‐carbon electricity assumptions represent a baseline operational reality for modern, target‐driven biomanufacturing facilities globally, rather than a regional exception.

Second, updates in leading life cycle assessment (LCA) databases have led to a drastic re‐evaluation of emission factors for plastic production (Berg and Carus 2024).

These changing framework conditions create a compelling need to reassess the sustainability balance of SUT. In light of ambitious net‐zero targets within the Scientific Based Target initiative (SBTi) to which many pharma companies have committed, a detailed understanding of these impacts is a strategic necessity (Booth et al. 2023). This study moves beyond high‐level estimates to create a granular, bottom‐up model of the SUT carbon footprint to identify and evaluate data‐driven mitigation strategies.

## 2 Methods and Materials

### 2.1 Overall Study Design and Functional Unit

**Figure 1:**
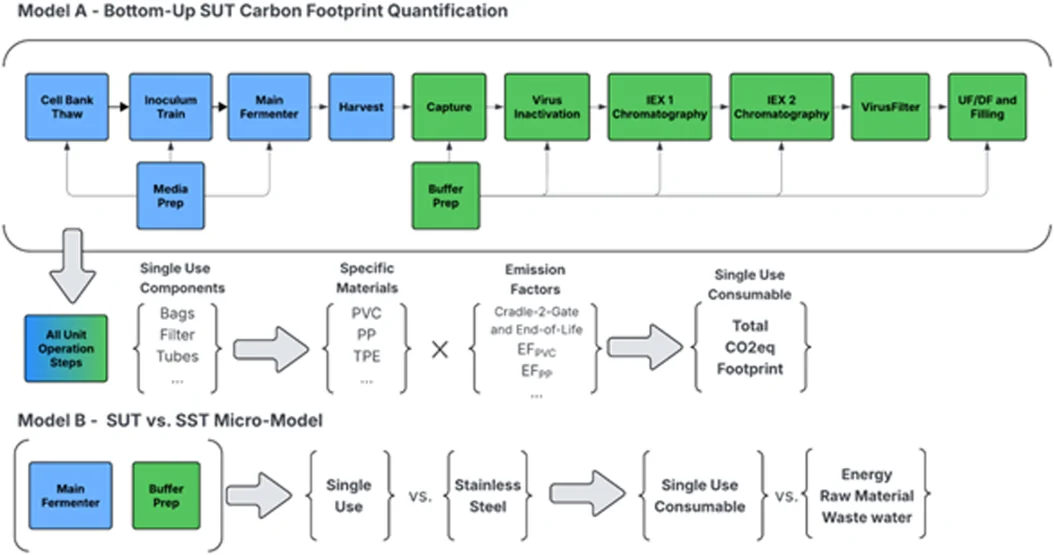
Schematic representation of the two‐part study methodology. Model A (top): The “Bottom‐Up SUT Footprint Model” maps the complete material flow for a 2000 L drug substance batch (blue: cell culture, green: purification). It quantifies the carbon footprint of all single‐use consumables (bags, filters, tubes) across all unit operations from cell bank thaw to bulk filling, based on physical disassembly and weighing (“cradle‐to‐gate” + EoL). Model B (bottom): The “SUT vs. SST Micro‐Model” provides a direct comparison of two specific unit operations (Main Fermenter and Buffer Preparation). It contrasts the Scope 3 material emissions of Single‐Use Technologies (SUT) against the Scope 2 energy emissions (steam, electricity) and water consumption of Stainless Steel Technologies (SST).

To accurately re‐evaluate the sustainability of SUT, this study employs a two‐part methodology— shown in Figure 1. First, a “Bottom‐Up SUT Footprint Model” (Model A) was developed to quantify the total carbon footprint of all single‐use consumables across different, real‐world facility archetypes. This model addresses exclusively the material‐related Scope 3 emissions and its results are presented in Sections 3.1 and 3.2. Second, a focused “SUT versus SST Micro‐Model” (Model B) was created to isolate and compare the core environmental trade‐off: the Scope 3 (plastic) emissions of SUT versus the Scope 2 (energy) emissions of SST for specific, interchangeable process steps. The results of this direct comparison are presented in Section 3.3.

The functional unit for Model A is defined as *all single‐use consumables and their packaging required for the manufacturing of one 2000 L scale drug substance batch*, from thaw of the cell culture vial until bulk filling. For Model B, the functional unit is the *execution of one specific unit operation* (here 2K bioreactor run or buffer preparation).

### 2.2 Model A: Bottom‐Up SUT Carbon Footprint Quantification of Case Studies

To generate high‐resolution data, a detailed, physical quantification protocol was applied. Three different models at the 2000 L scale were analyzed, representing a spectrum of facility designs:

1. “2K SU Benchmark (theoretical)”: A generic, theoretical model based on publicly available data of the NIIMBL ontology, serving as an industry benchmark (NIIMBL 2025).
2. “2K Full‐SUT (case study)”: A real‐world commercial process run in a facility designed almost entirely around single‐use systems at a Roche site in Basel, Switzerland. As this manufacturing process included two parallel cell culture trains for a bispecific antibody, its data has been normalized to a single train to ensure comparability with the other single‐train archetypes.
3. “2K Hybrid‐SUT (case study)”: A real‐world clinical process run in a hybrid facility at a Roche site in Penzberg, Germany, that combines SUT with significant SST components. The selected manufacturing process from clinical production represents the current technology platform.

The bottom‐up protocol comprised four stages: (1) extraction of the Bill of Materials (BOM), (2) verification of actual consumption by cross‐referencing with operational data and expert interviews, (3) physical disassembly of a representative set of all consumables, including packaging, into their individual components, and (4) identification of the respective polymer type and precise weighing of each component. This approach ensures the highest degree of accuracy and relevance to the specific operational procedures.

#### 2.2.1 Carbon Footprint Calculation of SUT in Model A

##### 2.2.1.1 System Boundaries

The analysis was carefully scoped to isolate the impact of the plastic consumables themselves as well as their packaging. Our methodology is directly guided by DIN EN ISO 14067 (Carbon footprint of products).

Included:

The analysis encompasses:

1. Upstream material emissions (“cradle‐to‐gate”): emissions from raw material extraction, manufacturing of specific precursors, and processing into the final consumable. This boundary specifically represents the supplier's delivery gate.
2. Downstream emissions (end‐of‐life, EoL): emissions from the end‐of‐life treatment (incineration), which were consistent for both investigated manufacturing sites.

This combined approach tracks the material‐related CO2eq impact from production to disposal, focusing on the consumables' Scope 3 burden. In the scope of the analysis were bags, filters, tubes, connectors, manifolds, and kits of all unit operation steps from seed train until drug substance bulk filling.

Exclusions and limitations: to focus specifically on the material‐related variable, several secondary parameters were excluded, such as facility‐level HVAC loads or the logistics chain to the manufacturing site. This includes on‐site post‐production autoclaving for solid SUT waste decontamination prior to disposal was excluded—as it is currently required only in the EU. Because these omitted parameters represent distinct carbon burdens that apply exclusively to the SUT lifecycle, their exclusion does not imply equivalence or a “canceling out” with SST operational utility inputs. Instead, this boundary definition systematically understates the SUT footprint, deliberately favoring the SUT scenario and establishing a highly conservative lower bound for its total carbon impact.

Excluded were also pre‐packed chromatographic columns, as these are usually reused over multiple cycles, depending on the phase of a product.

##### 2.2.1.2 Emission Factors

Specific suppliers' emission factors are typically proprietary and undisclosed. Consequently, our approach relies on market average material data sets from the Ecoinvent database for commodity plastics (Rest‐of‐the‐World/Global averages; v3.12). Given that quality and regulatory purity requirements for pharmaceutical‐grade plastics are substantially higher, the specific precursors utilized by SUT suppliers are likely to carry a higher carbon footprint; thus, our reliance on commodity averages represents a conservative baseline. SUT fabrication was modeled using granular processing emission factors (e.g., injection molding at 1.25 kg CO2eq/kg and pipe extrusion at 0.403 kg CO2eq/kg). Additional processing burdens, such as intermediate transport or gamma‐irradiation sterilization, were explicitly excluded to avoid geographical ambiguity regarding supplier locations and to account for the limited availability of high‐quality inventory data for commercial irradiation processes.

Due to the structural architecture of co‐extruded multilayer films and the proprietary boundary conditions of supplier material data sheets, a physical separation of the sub‐micron bag layers (e.g., PE, EVOH, and PA) was not feasible. Consequently, the total mass of the multilayer bag films was modeled by applying an average of the production emission factors of 20 representative plastics (ranging from low‐impact PE to high‐performance specialty polymers such as polysulfone). For end‐of‐life (EoL) treatment, a generic “mixed plastics” incineration data set from the Ecoinvent database (v3.12) was utilized. This approach serves as a robust and standardized LCA proxy, effectively balancing the higher manufacturing carbon footprint of specialty barrier polymers against their structurally altered stoichiometric carbon release during combustion, thereby ensuring a reliable and balanced representation of the consumable film footprint.

In total, the real emission factors of consumables are expected to be higher than considered in this paper, meaning that the CO2 footprint for SUT should be considered a conservative lower bound.

### 2.3 Model B: SUT vs. SST “Micro‐Model” Comparison

To test this paper's central hypothesis—the inversion of the SUT versus SST carbon footprint—we created a focused “micro‐model” comparing two representative unit operations. This approach moves beyond a full LCA to isolate the core environmental trade‐off: the Scope 3 (plastic) emissions of SUT versus the Scope 2 (energy) emissions of SST for sterilization (SIP/CIP).

Selected were (1) the 2000 L bioreactor run and (2) a 1200 L buffer preparation step to represent both core process steps and large‐volume ancillary support steps. Both full SUT and full SST configurations are used at the hybrid facility within the same environment, enabling a direct, controlled real‐life comparison.

This model allows us to quantify the “paradigm shift” at a granular level. We analyzed the operational procedures and applied the site's market‐based energy emission factors (per GHG protocol), which reflect significant renewable energy procurement. This ensures the model tests our re‐evaluated Scope 3 plastic footprint against a decarbonized, modern Scope 2 energy footprint.

The following model boundaries were deliberately set to focus only on this fundamental SUT versus SST differential:

Included in SUT model:

- “Cradle‐to‐gate” and end‐of‐life CO2eq emissions for all single‐use consumables identified in the bottom‐up analysis (as described in 2.2.1). In addition, we consider here defects at a rate of 11% for the bioreactor and 2% for the mixing bag: this rate is based on internal data from two production sites over a 3‐year period and reflects practical experience with material defects such as perforations in the film, fractured stirring blades, or foreign matter contamination. The defect consumables are directly disposed of. While these defect rates are specific to the sites analyzed, they highlight the practical necessity of accounting for material failures in sustainability assessments, a factor often overlooked in theoretical models.
  Included in SST model:
- CO2eq emissions for the thermal energy (steam) required for sterilization‐in‐place (SIP), and from the production of purified water and NaOH for cleaning‐in‐place (CIP) operations. This is considered the primary, differentiating energy driver for SST. In addition, waste water and the amortized production footprint of the steel vessel (assuming a 25‐year lifespan with 10 runs per year) were added.
  Intentionally excluded from both models (common basis): To focus the model on the key differences, the following emission sources were intentionally excluded, as they are considered comparable for both technologies or outside the defined operational scope:
- HVAC and cleanroom energy: operational HVAC energy was excluded from the system boundaries as a common baseline. In alignment with modern regulatory standards (e.g., EudraLex Annex 1), cleanroom zoning is determined by process closure rather than equipment material. Since modern SST facilities utilize functionally closed operations, both manufacturing configurations routinely operate under identical Grade D (ISO 8) conditions. Furthermore, commercial‐scale SUT setups do not inherently reduce facility footprints due to significant consumable warehousing and horizontal floor space requirements. Maintaining HVAC parameters outside the system boundaries, therefore, represents a conservative baseline choice that avoids confounding facility‐scale variables, a comparison further complicated by site‐specific technologies, for example, heat recovery integrated into the building's district heating network at certain locations.
- Operational energy (mixing, pumping): The energy required for core process actions (e.g., stirring, pumping, heating) is assumed to be equivalent for both technologies.
- End‐of‐life (EoL) credits: EoL impacts, such as CO2 credits from recycling steel or energy generation from burning waste, were excluded to maintain a strict “cradle‐to‐gate” comparison of the operational inputs.
- Waste pretreatment and transport: autoclaving of waste (SU disposables, filters needed for both) and waste transport to the incineration site were excluded.
- Operational contamination events and cleaning reworks were excluded from both the SUT and SST system boundaries. Unlike SUT material defects, which result in the immediate disposal of physical consumable mass prior to processing, SST asset operations present no equivalent per‐batch material scrap mode. Furthermore, because automated CIP/SIP sequences are highly validated and monitored via real‐time inline analytics, routine cleaning failures or sterility‐breach reworks are extremely rare (< 1% in routine operations) and exert a negligible impact on the overall lifecycle carbon footprint.
- Steel for manifold to operate SUT, as the impact was considered negligible over the equipment lifetime.

## 3 Results

### 3.1 Overall Impact of Single‐Use Consumables

**Figure 2:**
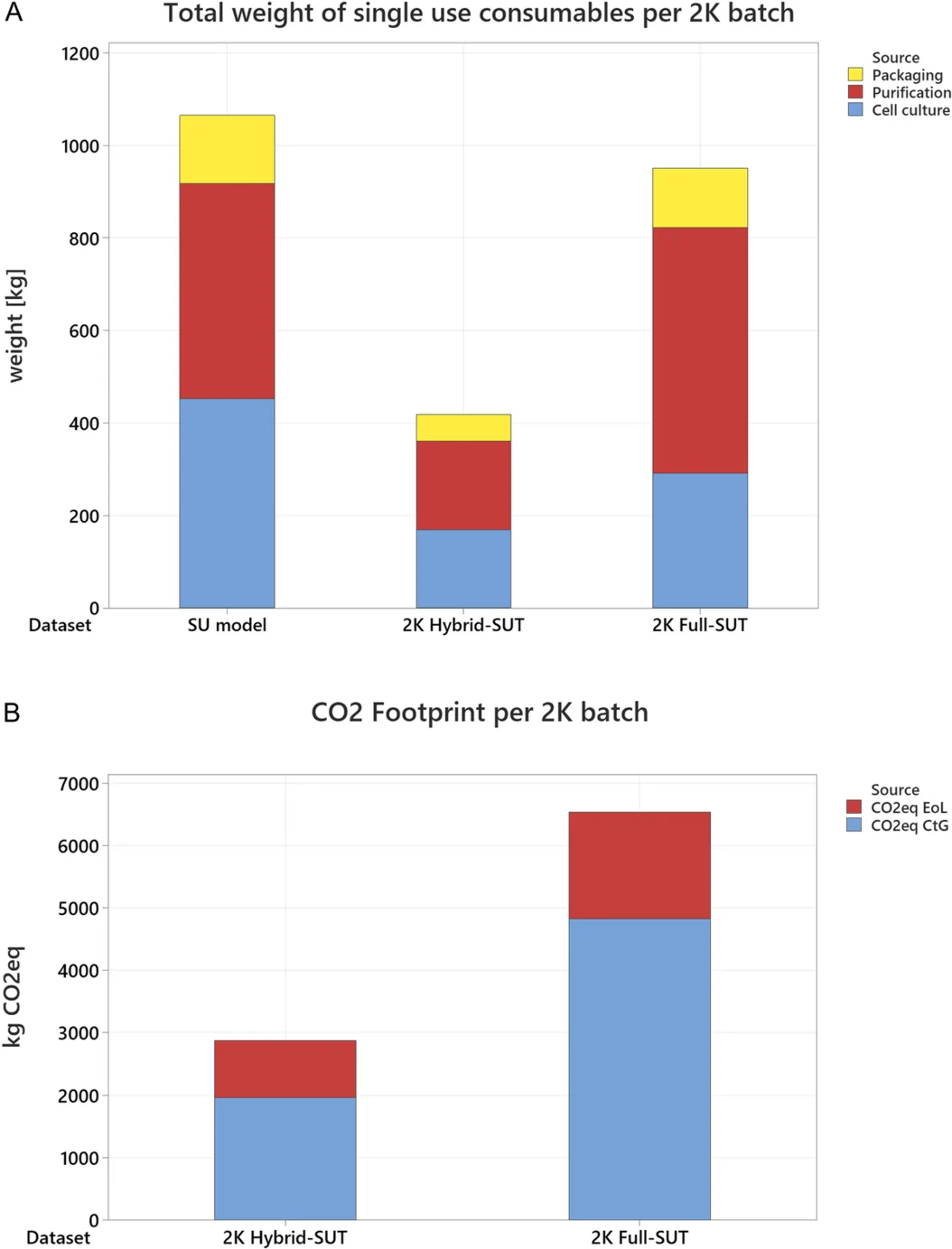
Quantification of material mass and carbon footprint per 2000 L batch. (A) Total mass of single‐use consumables. The chart compares the theoretical benchmark (SU Model) with the real‐world “Hybrid‐SUT” and “Full‐SUT” case studies. Colors indicate the process stage: cell culture (blue), purification (red), and packaging (yellow). The Hybrid model requires significantly less material mass (419 kg) compared to the Full‐SUT model (951 kg). (B) Carbon footprint (CO2eq). Translation of mass into carbon equivalents showing “cradle‐to‐gate” emissions (CtG, blue) and end‐of‐life incineration (EoL, red). The Full‐SUT process results in 6.5 tons of CO2eq, whereas the Hybrid‐SUT process generates 2.8 tons, illustrating the impact of strategic stainless steel integration.

The analysis in model A quantifies the SUT use per batch at 360 kg for the “2K Hybrid‐SUT” process and 822 kg for the normalized “2K Full‐SUT” process (Figure 2A). This corresponds to a carbon footprint of 2,9 and 6,5 tons CO2eq, respectively (Figure 2B). The data show a clear difference between the fully disposable “2K Full‐SUT” model and the “2K Hybrid‐SUT” operational model. Based on the extrapolation from the subset of manually weighed consumables and respective packaging, on average, 16% of the net weight of consumables needs to be added as the weight of packaging materials.

Our data on SUT weights for the “2K Full‐SUT” process is similar to the benchmark SU model in Figure 2A as a reference, confirming our quantification approach. It aligns as well with the values reported by Li et al. (2025) when the scope of analysis is harmonized. Li et al. included single‐use film/bags, ports, connectors (⅓ of our quantity), but excluded tubings, filters, and packaging—which contribute a significant additional amount of materials and waste (missing ⅔ of the quantity). Similarly, our SUT data shows 29% more mass than the assumption in the last full life cycle assessment (LCA) of 2K SUT used for their calculation (Budzinski et al. 2022), indicating that plastic quantities in SUT have been underestimated so far.

The “2K Hybrid‐SUT” process footprint is 62% lower than the “2K Full‐SUT”. This underscores that the strategic integration of SST is a decisive factor for emissions reduction. The primary drivers for this significant reduction are found in the targeted substitution of high‐mass consumables, as the following hotspot analysis reveals.

### 3.2 Hotspot Analysis: Identifying the Main Drivers

**Figure 3:**
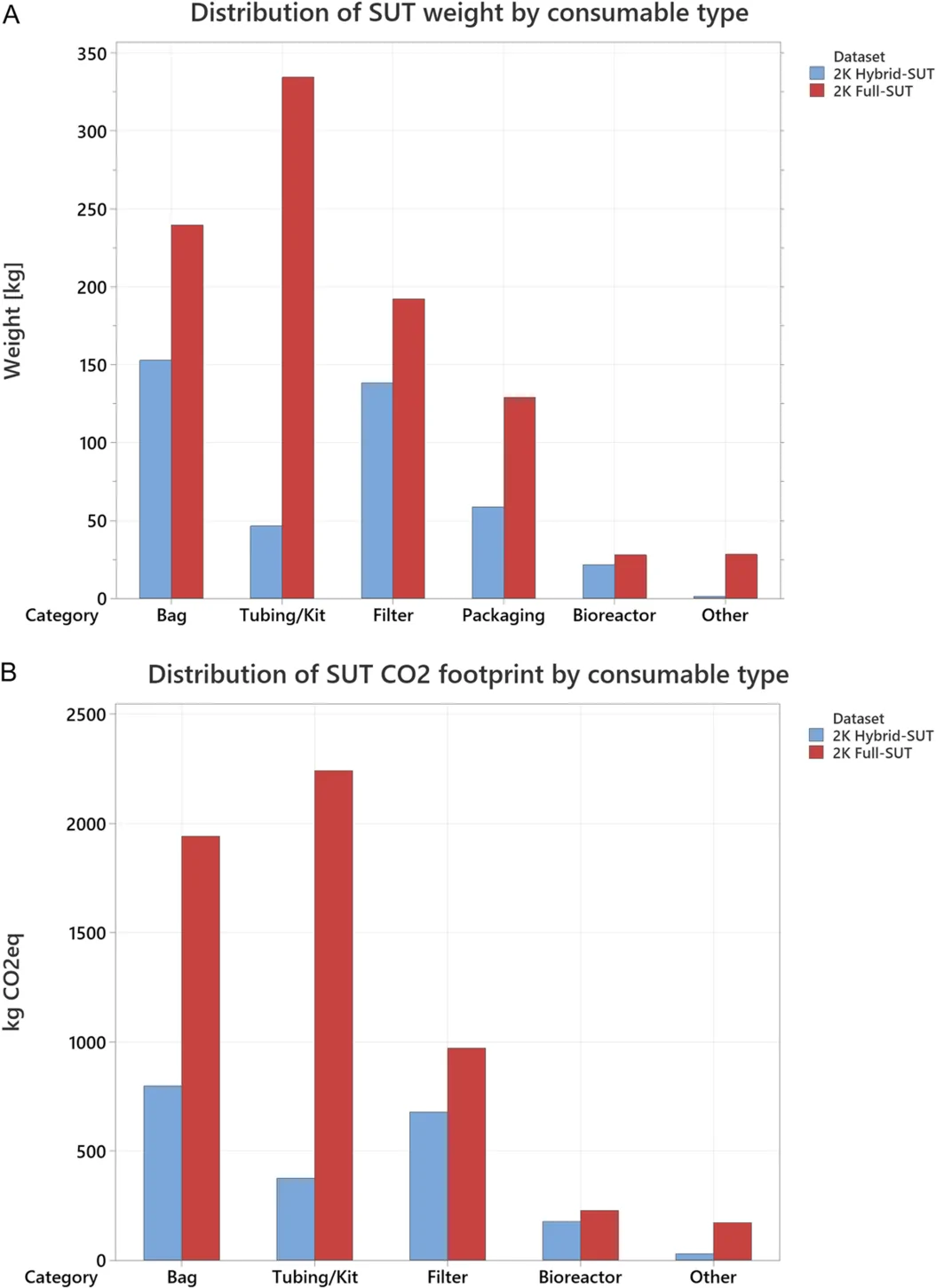
Hotspot analysis of single‐use consumables. (A) Weight distribution. Breakdown of consumable mass by category for both facility archetypes. Tubing/kits and bags are the dominant contributors to weight in the Full‐SUT model. (B) CO2eq distribution. The corresponding carbon footprint reveals that bags and tubing/kits are the primary emission drivers. Note that while filter weight is comparable between archetypes (due to depth filters), the Full‐SUT model shows a drastic increase in emissions from bags and tubing due to the reliance on large‐volume disposable containment and transfer lines.

The analysis shows that tubing/kits, bags, and filters are the largest hotspots, together accounting for the majority of the plastic mass and associated emissions (Figure 3).

A Pareto analysis of the sub‐categories of the two archetypes reveals that the greatest reductions in the “2K Hybrid‐SUT” process are achieved by eliminating or significantly reducing mass‐intensive but low‐complexity consumables, particularly large buffer bags > 1000 L volume and long tubing lines.

The quantity of filters is in a similar range for both archetypes. The majority of the filter weight stems from depth filters, which are used not only for harvest clarification but also within purification to prevent filter blockages. Although the filter material itself (e.g., cellulose) has a lower emission factor, the substantial mass of the surrounding plastic caskets and capsules contributes significantly to the overall CO2 footprint.

### 3.3 Direct Comparison: Single‐Use vs. Stainless Steel in Model B

The analysis of the hybrid facility (3.1) and its hotspots (3.2) demonstrated that the strategic retention of SST for high‐volume operations is the key driver of its lower carbon footprint. This leads to the central question of this study: What is the direct, quantitative trade‐off between SUT and SST for these specific, interchangeable operations?

**Figure 4:**
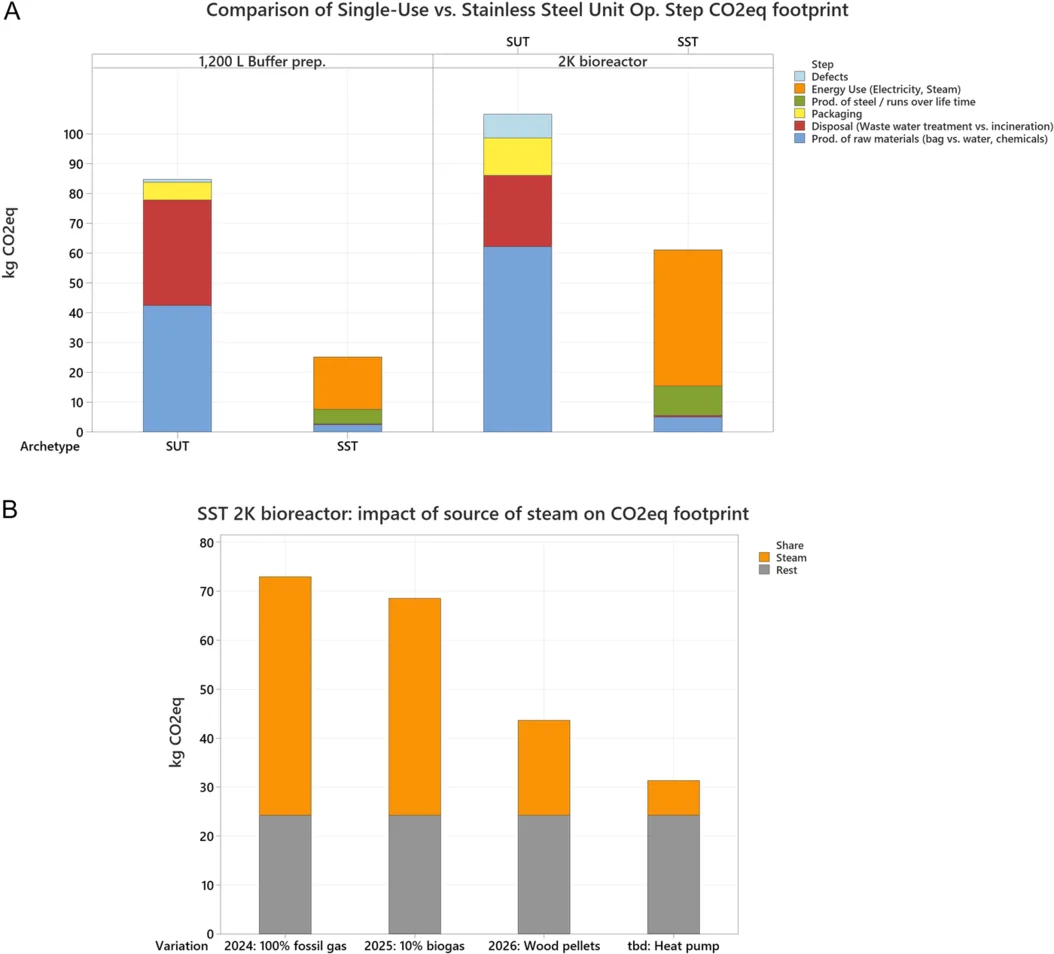
Direct comparison of environmental impacts (micro‐model). (A) SUT vs. SST comparison. Life cycle assessment of two representative unit operations: a 1200 L buffer preparation step and a 2000 L bioreactor run. The bars show that the material‐related emissions of SUT (blue/red) significantly exceed the energy‐driven emissions of SST (orange), primarily steam. Defects (light blue) are included for SUT. (B) Sensitivity analysis of steam sources. Impact of decarbonizing the energy supply for the SST 2K bioreactor, using the reported emission factors of the site Penzberg. While current fossil‐gas‐based steam (2024) already outperforms SUT, the transition to biogas (2025), and in the near future wood pellets (2026) will further widen the sustainability gap in favor of stainless steel. Preliminary calculations with an industrial heat pump at COP 2.5 using current market‐based electricity emissions can reduce these emissions even to ca. 37%.

To answer this, our direct “micro‐model” comparison (Model B as defined in 2.3) was applied. The results show that SST operations can have less than half the CO2 emissions of their SUT pendants (Figure 4).

For SST, steam for the SIP process is the largest driver (87% for the 2K bioreactor). The consumption of NaOH, water, and electricity calculated with market‐based emission factors, has a very low impact on the CO2eq emission. Even with a pessimistic assumption (pure fossil gas for steam generation), the advantage for SST remains under current conditions. On the other hand, future technical investments at the site level into more sustainable steam production (e.g., biomass or industrial heat pumps) can significantly reduce the emission of SST even further (all in Figure 4B).

For SUT, the manufacturing of the consumable itself is the largest lever, alone causing more emissions than the entire amortized SST process step, including CIP/SIP operation. Even with optimistic assumptions, such as −30% emission factor due to specific supplier data or −50% reduction for plastic waste via chemical pyrolysis, the SUT footprint merely meets the SST value.

## 4 Discussion

This study provides a granular quantification of the CO2 footprint of SUT and demonstrates that established assumptions about sustainability must be re‐evaluated.

### 4.1 The Inversion of the SUT vs. SST Footprint

A comprehensive LCA by Budzinski et al. (2022) established a key benchmark for 2000 L SUT processes. Using 2021 data, it identified energy consumption (45,233 kWh) as the dominant CO2 driver, representing 89% of the total footprint of a batch. With the location‐based U.S. grid factor of 424 g/kWh of 2021, this corresponded to 19.2 tons of CO2eq. The SUT consumables themselves were considered a minor contributor, accounting for only 7.5% (an implied 1.6 tons CO2eq) based on a SUT mass of 769 kg.

The established sustainability paradigm for SUT must now be fundamentally re‐evaluated. Our findings reveal that both drivers have fundamentally shifted. First, the energy penalty of SST is largely outdated. While improvements in “location‐based” grid CO2e intensity play a role, ‐ as outlined in the Introduction ‐ the primary driver is the active, large‐scale procurement of renewable energy to meet ambitious SBTi targets.

To quantify the impact of these procurement decisions, the “market‐based” accounting method—specified in the GHG Protocol's “Scope 2 Guidance” (Sotos 2015)—is the requisite mechanism. As confirmed by Booth et al. (2023), 100% of major pharmaceutical companies utilize renewable energy procurement as a key decarbonization. Recent analysis by Stachelscheid and Dutzi further underscores that the market‐based approach is the essential lever enabling companies to demonstrate these individual decarbonization efforts independent of local grid mixes (Stachelscheid and Dutzi 2025). Consequently, despite existing heterogeneity in disclosure practices, the market‐based method remains the indispensable metric for tracking progress against net‐zero targets in this analysis.

Applying a real‐world market‐based emission factor of 77 g CO2e/kWh, reflecting 2025 operational data from the Roche Penzberg site, to the energy consumption cited by Budzinski et al. slashes the energy footprint by 82% to just 3.5 tons of CO2eq. This dramatic reduction is possible because energy is a decarbonizable resource. In sharp contrast, the “cradle‐to‐gate” emissions for fossil‐fuel‐based plastics are chemically fixed. Furthermore, this dynamic introduces a critical temporal dimension to facility design and investment cycles already today. Biopharmaceutical manufacturing facilities typically require multi‐year timelines for planning, construction, and GMP commissioning. Consequently, even if a full‐SUT facility is conceptualized today within a region characterized by a historically carbon‐heavy local grid, the global trajectory of energy transition assures that the local grid mix will have substantially shifted toward lower carbon intensities by the time routine operational manufacturing commences. This planning‐to‐operation lag renders static, legacy energy assumptions from foundational SUT LCAs (e.g., Pietrzykowski et al. 2011 or Budzinski et al. 2022) obsolete for prospective sustainability assessments, as they neglect the baseline operational reality of the coming decades.

Second, this study demonstrates that the “Scope 3” footprint of plastics is far larger than previously assumed. Our bottom‐up analysis shows that consumable plastic mass per batch ranges up to 822 kg (full disposable SUT facility) plus 128 kg packaging waste‐ significantly higher than those reported in previous LCAs (Pietrzykowski et al. 2011 or Budzinski et al. 2022). On top comes a fundamental shift in emission drivers. The updates of emission factors in the Ecoinvent database from v3.9 to v3.12, which now better account for fugitive methane emissions from natural gas and oil extraction, have drastically increased the “cradle‐to‐gate” footprint of plastics (Stoikou et al. 2025; FitzGerald et al. 2023, 2024; FitzGerald and Sonderegger 2022). The Renewable Carbon Initiative (RCI) assessed that the footprint of commodity plastics has increased by around 30% (Berg and Carus 2024). Our specific process inventories directly reflect this macro‐trend; for instance, the emission factor for low‐density polyethylene (LDPE) granulate {RoW} rose by +32% from 2.48 kg CO2eq/kg in v3.9 to 3.30 kg CO2eq/kg in v3.12, while ethylene vinyl acetate (EVA) copolymer {RoW} surged by 34% from 2.52 to 3.37 kg CO2eq/kg. At the same time, these data points expose vital regional disparities, with the respective European datasets in v3.12 standing significantly lower at 2.41 kg CO2eq/kg for LDPE (−3% in comparison to v3.9) and 2.83 kg CO2eq/kg for EVA (+12%)—underscoring that supply chain geography is becoming a critical differentiator in SUT sustainability profiling. The same reevaluation rooted in the limitations of legacy Ecoinvent data sets are happening in other industries, for example, in the textile industry the use of polyester may have been underestimated by up to 44%—skewing the LCA results and sustainability labels (Nautiyal et al. 2025).

Our bottom‐up analysis of SUT consumables, including packaging, results in a footprint of 7.6 tons of CO2eq per batch. This footprint now appears to be substantially larger—potentially twice as much—than the market‐based SUT energy footprint calculated above. This comparison strongly suggests that the paradigm has inverted. Furthermore, as established in our methodology, this 7.6 ton value represents a conservative lower bound. The omission of specific life‐cycle burdens unique to SUT, such as supplier‐side gamma sterilization, pharma‐grade plastic emission factors, supply chain, and post‐production solid waste autoclaving, means that the actual environmental deficit of full‐disposable bioprocessing is likely even more pronounced in commercial practice. This conservative nature is further illustrated when benchmarking our granular boundaries against historical LCA frameworks. While our bottom‐up model isolates strict component manufacturing mechanics (averaging 0.4–0.5 kg CO2eq/kg for molding and extrusion), a simplified framework developed by Ramasamy (2018) utilized an aggregated fabrication proxy of up to 1.9 kg CO2eq/kg to encompass bundled supplier‐side transport, cleanroom assembly, and gamma sterilization. If these additional life‐cycle components were fully integrated and scaled across our quantified consumable mass of 822 kg for a full‐SUT batch, it would add an estimated 1.2 tons of CO2eq emissions to the footprint. This highlights that the material‐related environmental burden, not energy, is the new hotspot for SUT bioprocessing and must be the focus of future sustainability efforts. A new, complete LCA is needed to confirm these findings.

For an industry and its supply network already committed to ambitious SBTi targets, successfully achieving these goals is now contingent on developing effective strategies to mitigate precisely these material‐related Scope 3 emissions. Ultimately, this highlights a pressing need for collective action. We call upon single‐use suppliers to dismantle proprietary data barriers and openly disclose component‐specific emission factors. Granular transparency regarding production locations, plastic precursor sourcing, renewable energy adoption, and site‐specific sustainability initiatives is the only viable foundation for verifiable lifecycles and true, sector‐wide decarbonization.

### 4.2 The Hybrid Model as an Effective Mitigation Strategy

The “2K Hybrid‐SUT” case study proves to be a highly effective mitigation strategy, although this configuration did not arise from an active sustainability decision, but rather evolved pragmatically over time to optimize operational workflows using existing infrastructure.

Our results show that this hybrid model has a significantly lower footprint (2.9 tons CO2eq) compared to the “Full‐SUT” model (6.5 tons CO2eq) (Figure 2B). The hotspot analysis (3.2) provides the reason for this: the hybrid facility's footprint is lower simply because it *avoids* the primary hotspots of the Full‐SUT model, namely the mass‐intensive large‐volume buffer bags (≧ 1000 L) and their associated long tubing lines.

For the upstream process, large‐volume media is prepared in existing stainless steel vessels and transferred via online sterile filtration directly into the bioreactor system or into smaller SUT satellite bags as needed. This direct‐transfer architecture yields significant plastic savings by completely eliminating the need for intermediate large‐volume single‐use holding bags and their associated complex transfer tubing assemblies. Using a 2000 L steel harvest tank instead of a single‐use harvest bag saves an additional 10 kg of plastic, equivalent to 67 kg CO2eq

For the downstream purification steps, buffer concentrates are combined with inline dilution. Concentrated buffers (e.g., 10×) are prepared in SST vessels and then filtered into small, manageable SUT bags (e.g., 200 L). This practice has multiple benefits: (1) it replaces > 1000 L SUT bags with smaller volume bags, and (2) these small bags remain physically mobile (on wheels), which completely eliminates the need for extensive, heavy single‐use transfer tubing assemblies and complex distribution manifolds from distant preparation rooms. Given that our bottom‐up hotspot analysis identified these long‐distance transfer tubings as a major polymer mass contributor—accounting for 71% of the total tubing category weight—this logistical layout change provides a massive secondary plastic‐saving benefit. (3) The purified water bags needed for dilution can be reused multiple times within strict validation and expiry limits. The hybrid facility also implemented other pragmatic solutions, like reusable stainless steel connectors or reusing transfer kits within the same product campaign under validated expiry windows, thereby all contributing to its lower CO2eq footprint.

All these measures are directly validated by our “micro‐model” analysis (3.3): the micro‐model (Figure 4) quantified the high impact of these specific SUT components, showing that replacing just a single 1200 L buffer prep step saves ~66 kg CO2eq. The “Hybrid‐SUT” case study, therefore, demonstrates that a pragmatic “fit‐for‐purpose” hybrid solution is the most effective and immediately available mitigation strategy to reduce the CO2 footprint. It resolves the central conflict of goals: it pragmatically leverages the CO2 advantages of SST for high‐volume operations without sacrificing the operational advantages of SUT (such as flexibility, lower CAPEX, and rapid product change‐over) for the core process steps. While certain multi‐batch reuse scenarios for SUT bags are technically feasible within strict validation barriers—and are already fully integrated into the baseline boundaries of our SUT model—the structural elimination of transfer lines via facility layout optimization remains a far more impactful lever for total plastic mass reduction.

Therefore, the true key to sustainability is the underlying principle: the systematic avoidance of high‐volume consumables. The use of concentrates and inline dilution is the most powerful example of this, as it can be equally applied to optimized full‐SUT designs to significantly reduce plastic waste.

### 4.3 Solutions for More Sustainable SUT Use

The comparative analysis between a process in a full disposable facility and a process in a hybrid facility revealed that more than 50% of the single‐use plastic‐related carbon footprint can already be avoided by systematically implementing existing technologies. This suggests a “law of diminishing returns” for full SUT implementation. While the operational flexibility of SUT remains valuable for the complex core process (bioreactor, chromatography), the environmental costs of disposable plastics outweigh the benefits for simple, high‐volume auxiliary operations.

**Figure 5:**
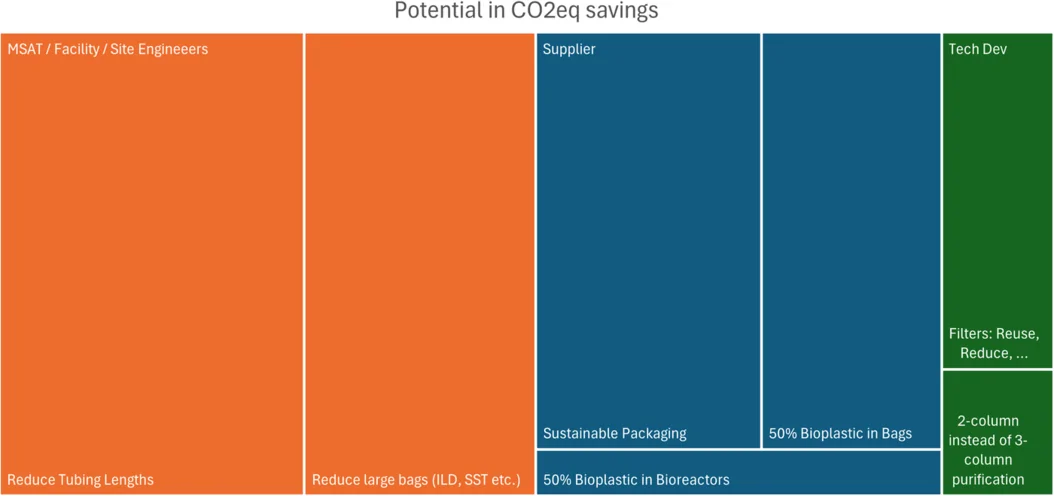
Strategic mitigation potential by stakeholder. A tree map illustrating the estimated CO2eq savings potential categorized by area of responsibility. The largest levers lie with MSAT/facility engineers (orange) through the implementation of hybrid designs (reducing large bags/tubing) and suppliers (blue) through sustainable packaging and bioplastics. Technical development (green) contributes by optimizing processes to reduce filter and unit operation steps.

Further opportunities have been captured in the BioPhorum environmental sustainability roadmap 2022 (Hamid et al. 2022), and many suppliers report on working on these key actions. Although emerging technologies like bioplastic and chemical pyrolysis of mixed plastic waste offer long‐term potential for a circular economy, without additional incentives, they currently face significant challenges regarding scalability, energy efficiency, and economic viability (Dai et al. 2024; Parveen et al. 2024). Therefore, short‐term mitigation strategies should focus on waste reduction (“Reduce”) and reuse. Our findings indicate areas of high impact for all stakeholders. The following sections describe for each level the solution areas within their accountability and area of control, with their potential represented in Figure 5:

- Technical developers: The focus for product process development should not only be on titer and yield but also on optimizing harvest clarification (Yeop et al. 2025) and capture step (Koehnlein et al. 2024) to reduce depth filter size and washing buffer volumes. Technical development can focus on further reuse options, for example, reusing depth filtration (Spensberger et al. 2025).
- Facilities/manufacturing science and technology (MSAT): Transfer the learnings from the hybrid facility outlined in this study: large volume buffer preparation in stainless steel tanks, stainless steel harvest tanks, usage of buffer concentrates with inline dilution, and to some extent, reuse of bags or kits can significantly reduce the CO2 footprint per campaign. This is not only achieved by bags with smaller volume, but also on the operational level when bags are still movable and less tubes are needed.
- Site engineers: Our findings show that reductions of Scope 1 and 2 emissions are direct benefits for the CO2 footprint of biomanufacturing. These can have direct near‐term impact, for example, up to 24% CO2eq reduction by optimizing the HVAC setup (M'Baye 2022) or long‐term by further integration of renewable energy or fossil‐free steam production at the site. The energy footprint can be further improved up to 90% with heat recovery of HVAC or SIP and low‐energy water purification (Beck et al. 2024). For new “greenfield” constructions of facilities, the strategic implementation of SST for large volume buffer and media preparation, and at least the option for CIP/SIP should be considered a primary sustainability measure.
- Suppliers: Transparency regarding specific emission factors, material of construction, and weights of individual product components is crucial to enable customers to make informed choices regarding recyclability. Redesigning disassembly options and reducing the amount of silicone tubes into preassembled consumables can help chemical pyrolysis, which is sensible to silicone contamination (Dai et al. 2024). Investing in product quality to reduce defects (> 10% for more complex consumables) improves sustainability, as well as exploring recyclates for packaging remain important areas for action and low‐hanging fruits, as our data indicates up to 14%–17% of emissions coming from packaging only.
- Future sustainability strategies must therefore weigh feasibility: while energy grids are decarbonizing rapidly, reducing the material footprint of registered products is far more complex, restricted by regulatory in GMP environment and the reliance on supplier innovation. Ultimately, this study demonstrates that achieving genuine sustainability in biomanufacturing is not about finding a single, permanent solution, but about embracing a dynamic process of continuous, data‐driven re‐evaluation to navigate a constantly evolving technological and ecological landscape.

## Funding

The authors have nothing to report.

## Ethics Statement

The authors have nothing to report.

## Conflicts of Interest

The authors declare no conflicts of interest.

## Data Availability

The data that support the findings of this study are available from the corresponding author upon reasonable request.

## 1

See Table A1

**Table A1:** Collected mass of SUT consumables in various categories. The data represents the 2K drug substance processes from two manufacturing sites, with slight variations due to facility layout or internal processes (e.g., batch size for media/buffer preparation due to demand).

Unit operation steps	Kategorie	SUT weights (kg)
Cell bank thaw	Bioreactor	0.2
	Tubes and connectors/kits and manifolds	3.5–5.4
Inoculum train	Air filter	0.1
	Bioreactor	11.6–17.4
	Filter	24.6 (if needed)
	Tubes and connectors/kits and manifolds	16.9–23.0
Main fermentation and harvest	0.1—0.2 µm filter	7
	Air filter	1.5
	Bioreactor	10.1
	Tubes and connectors/kits and manifolds	10–80
	Bag—large scale	up to 10
	Bag—mid scale	up to 38
	Depth filter/manifold	50–74
Capture and virus inactivation	0.1—0.2 µm filter	9–13.6
	Air filter	0.5
	Bag—small scale	up to 88
	Bag—large scale	9–156
	Bag—mid scale	up to 75
	Depth filter/manifold	up to 36
	Tubes and connectors/kits and manifolds	15–118
IEX 1/IEX 2	0.1—0.2 µm filter	up to 30
	Bag—small scale	7.0
	Bag—large scale	8.9
	Bag—mid scale	19.3–27.9
	Tubes and connectors/kits and manifolds	up to 26
Virus filtration	0.1—0.2 µm filter	6.2
	Nano filter/manifold	1.9
	Tubes and connectors/kits and manifolds	up to 41
UF/DF and filling	0.1—0.2 µm filter	up to 19
	Bag—small scale	up to 21
	Tubes and connectors/kits and manifolds	3.7
	UF/DF filter	2.8
Media/buffer	0.1—0.2 µm filter	up to 21
	Air filter	0.3
	Bag—small scale	up to 43
	Bag—mid scale	up to 36
	Tubes and connectors/kits and manifolds	up to 222
